# Molecular Evidence for Lessepsian Invasion of Soritids (Larger Symbiont Bearing Benthic Foraminifera)

**DOI:** 10.1371/journal.pone.0077725

**Published:** 2013-10-29

**Authors:** Gily Merkado, Maria Holzmann, Laure Apothéloz-Perret-Gentil, Jan Pawlowski, Uri Abdu, Ahuva Almogi-Labin, Orit Hyams-Kaphzan, Anna Bakhrat, Sigal Abramovich

**Affiliations:** 1 Ben Gurion University of the Negev, Beer Sheva, Israel; 2 Department of Genetics and Evolution, University of Geneva, Genève, Switzerland; 3 Geological Survey of Israel, Jerusalem, Israel; CSIR-National Institute of Cceanography, India

## Abstract

The Mediterranean Sea is considered as one of the hotspots of marine bioinvasions, largely due to the influx of tropical species migrating through the Suez Canal, so-called Lessepsian migrants. Several cases of Lessepsian migration have been documented recently, however, little is known about the ecological characteristics of the migrating species and their aptitude to colonize the new areas. This study focused on Red Sea soritids, larger symbiont-bearing benthic foraminifera (LBF) that are indicative of tropical and subtropical environments and were recently found in the Israeli coast of the Eastern Mediterranean. We combined molecular phylogenetic analyses of soritids and their algal symbionts as well as network analysis of *Sorites orbiculus* Forskål to compare populations from the Gulf of Elat (northern Red Sea) and from a known hotspot in Shikmona (northern Israel) that consists of a single population of *S. orbiculus*. Our phylogenetic analyses show that all specimens found in Shikmona are genetically identical to a population of *S. orbiculus* living on a similar shallow water pebbles habitat in the Gulf of Elat. Our analyses also show that the symbionts found in Shikmona and Elat soritids belong to the *Symbiodinium* clade F5, which is common in the Red Sea and also present in the Indian Ocean and Caribbean Sea. Our study therefore provides the first genetic and ecological evidences that indicate that modern population of soritids found on the Mediterranean coast of Israel is probably Lessepsian, and is less likely the descendant of a native ancient Mediterranean species.

## Introduction: The Lessepsian Invasion

**Figure 1:**
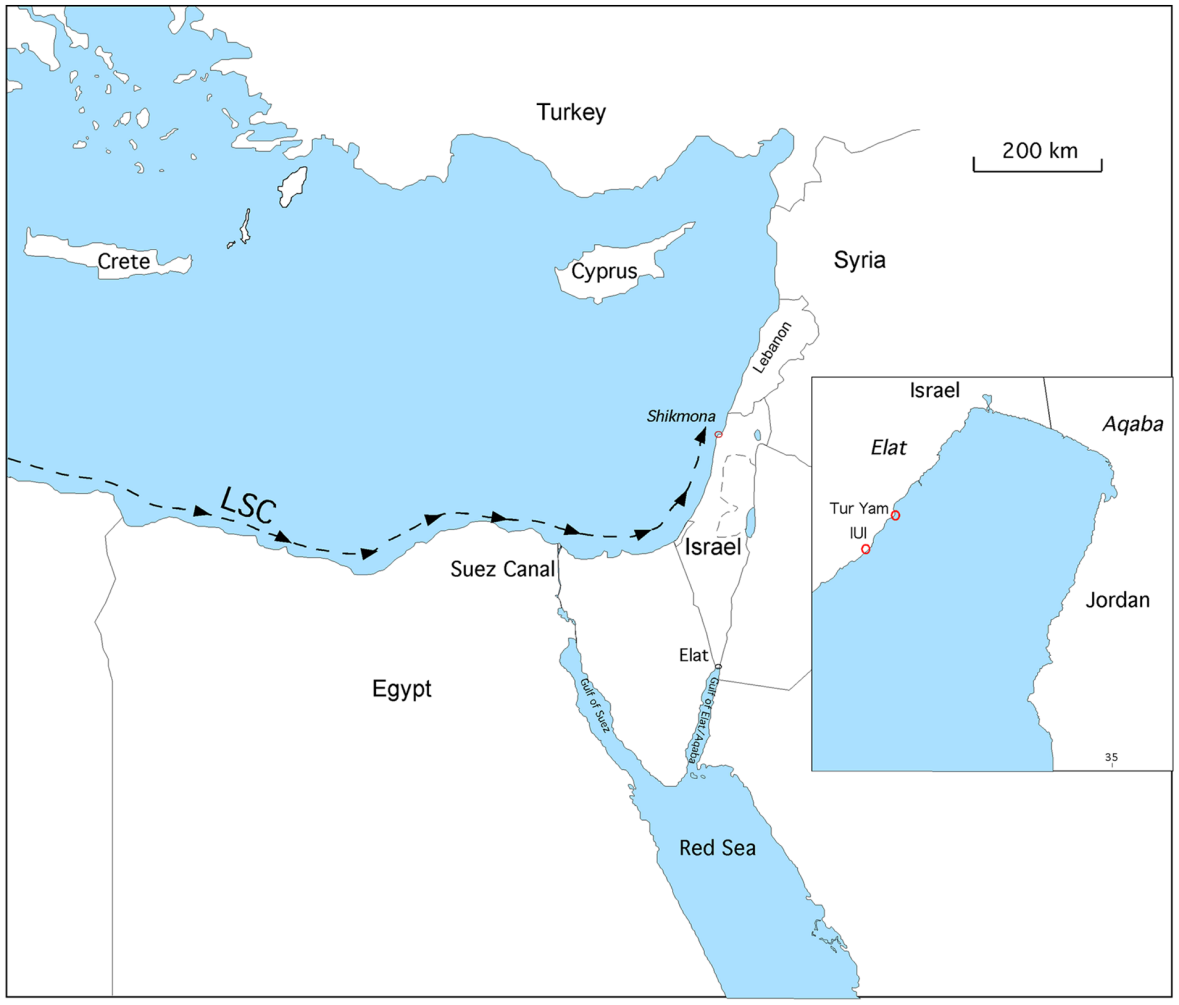
The two study localities in the Gulf of Elat, northern Red Sea represent different environments: Halophila stipulacea seagrass meadows at Tur Yam, and the shallow water environment with pebbles in front of the IUI station. In the Mediterranean, living specimens were studied from a known hotspot of soritids in Shikmona, Haifa. The longshore current dictates the anticlockwise distribution of the Lessepsian invaders.

In the last few decades, we have been witnessing a dramatic and rapid change in the composition of the marine biota of the eastern Mediterranean due to invasion of alien species [1]–[3]. The majority of the invasive species are tropical, of Indo-Pacific origin, which migrated from the Red Sea to the Mediterranean through the Suez Canal. These organisms, called Lessepsian migrants constitute a growing component of the biodiversity in the Eastern Mediterranean Sea. There are three main factors that promote this Lessepsian invasion phenomenon. First, the opening of the Suez Canal in 1869 that created an artificial connection between the Mediterranean and the Indo-Pacific realm via the Red Sea (Figure 1). Second the ongoing warming of sea surface temperatures in the last decades [4], [5]. This process enables tropical species to survive, mostly in the Eastern Mediterranean basin, with a potential to spread also to the western basin with the continuation of the warming trend. Third, the damming of the Nile River in 1965 by the high Aswan Dam in Egypt. The dam has blocked nutrients from discharging into the Eastern Mediterranean and created hyper-oligotrophic conditions there, suitable for many tropical species [6]–[8]. Until now more than 500 alien species have been reported, and many more are being discovered each year, making the Mediterranean a hotspot of marine bioinvasions [1], [3], [9]. The ecological, environmental and economic aspects of this phenomenon have received a great scientific interest in past few years (see discussion in [10]; www.ciesm.org/atlas).

The Lessepsian invaders have a significant impact on the species composition and the ecosystem in the receiving environment. Studies have shown that the invasive population can outcompete the indigenous population, and become dominant in a certain habitat (see examples in [11]). Other studies showed that the appearance of a new species might change the foraging patterns of certain species, due to the introduction of new prey [10], [12]. Introduction of new species to the Mediterranean, such as the jellyfish *Rhopilema nomadica* had a major impact on the fisheries, tourism and coastal installations [11]. Among the Lessepsian invaders, are larger symbiont-bearing benthic foraminiferal species (LBF) that often serve as sensitive marine bioindicators of ecological stress [13], [14]. LBF defines a group with shell size generally larger than 2 mm in diameter that are typical to warm and shallow oligotrophic tropical and subtropical oceans [13], [15]. Species of this group are known to respond to rising temperatures over a certain level in a similar way as corals, by losing their endosymbionts in a phenomenon known as bleaching [16], [17]. The tempo and scale of their invasion from the Red Sea into the Eastern Mediterranean provides a unique opportunity to evaluate the processes and mechanism that promotes tropical invasion of coral reef associated organisms. In this study, we used Red Sea soritids (LBF) that have recently invaded the Eastern Mediterranean as a model system for unique characterization of the ongoing Lessepsian invasion. Soritids are larger benthic miliolids that inhabit recent tropical and subtropical oligotrophic water, and are characterized by a large porcelaneous discoidal test and dinoflagellate symbionts belonging to the *Symbiodinium* species complex. In this study, we combined molecular phylogenetic analysis of the soritids and their algal symbionts to compare their populations from the Gulf of Elat, northern Red Sea and from the Mediterranean locality off Shikmona. Our data provide the first evidence on the source of the living populations of soritids in the Eastern Mediterranean coast.

## Methods

### The Study Area

#### The Eastern Mediterranean

The Eastern Mediterranean is defined by its extreme oligotrophy and higher salinity and temperature values: Summer sea surface temperatures (SST) reach a tropical value of 30°C with salinity as high as 39.7 [18], [19]. Over the past 44 years, an increase of >2°C has been recorded in the Eastern Mediterranean, most of which occurred since the 80’s. The rate of warming in the Mediterranean Sea, based on satellite data from 1990–2006, is 0.067°C per year, more than double the global forecasts [4], [20].

**Figure 2:**
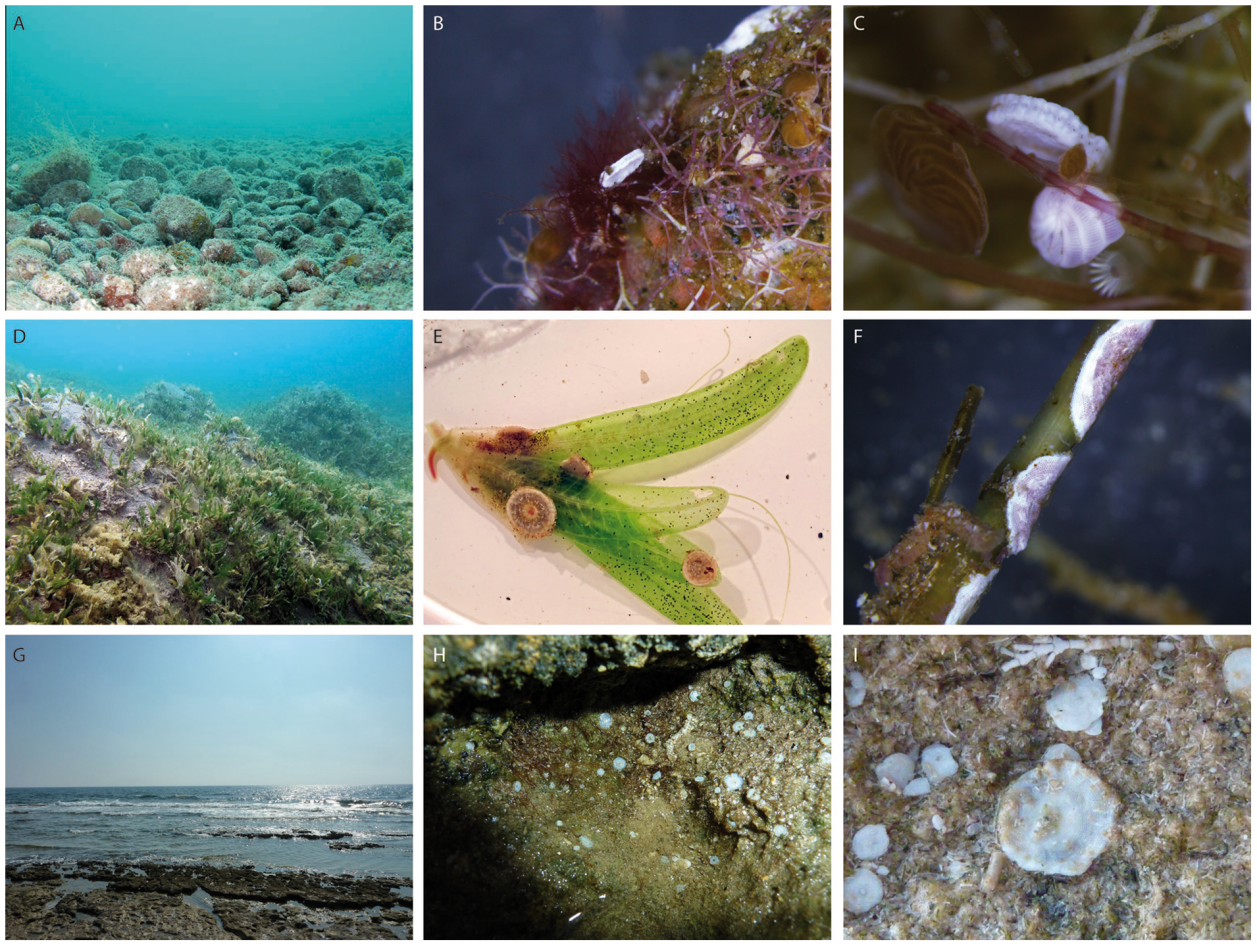
Habitat types of soritids in the Gulf of Elat and Shikmona. A–C. Pebbles and boulders at IUI station. A. Wide view of the habitat. B–C. Closer views of the algae that cover the pebbles. Note the specimen of S. orbiculus attached to the algae and the associated LBF species. D–F. Seagrass meadows (H. stipulacea) at Tur Yam. D. Wide view of the habitat. E. A. hemprichii attached to H. stipulacea leaves. F. Specimens attached to H. stipulacea stem. G–I Beach rock environment with pebbles at Shikmona. G. Wide view of the habitat; H–I. Closer view on the living specimens of S. orbiculus attached to the pebbles.

Soritids, namely *Sorites orbiculus* were once common in the Tethyan Mediterranean and were found in cores from Ashqelon (Southern Israel), in sediments from marine isotopic stage 9 and 8, 320-250 ka BP [21], [22]. This species was also found in Holocene cores (2 ka BP) taken from the inner harbor in Caesarea (Northern Israel) [23]. Recent soritids have been reported from various sites in the Mediterranean and have been considered as aliens [3], [8], [24], [25]. Living specimens of soritids are now found throughout the rocky environments in the region and forming a known hotspot in Shikmona (northern Israel, N32 49.450 E034 57.273), which was selected as the target area of this study. At this location specimens were collected from pebbles from abrasion platforms, at a water depth of ∼20 cm (Figures 1–2).

#### The Gulf of Elat

The Gulf of Elat is a morpho-tectonic branch of the Red Sea, which is a part of the Syrian African rift system. It is situated in the desert between the Sinai and the Arabian peninsulas, an area characterized by high evaporation rates due to high temperatures and dry air. There are minor seasonal fluctuations in the water temperature (20.5–27.4°C) and salinity (40.3–41.6). Dissolved oxygen values are close to 100% for the entire water column, and the gulf’s water is considered to be oligotrophic [26].

Soritids are abundantly found in the Gulf of Elat, represented by three morphospecies: *Sorites orbiculus* Forskål, *Sorites variabilis* Lacroix, and *Amphisorus hemprichii* Ehrenberg, genetically assigned to three phylotypes with some additional branching of *S. orbiculus* [27]. They occupy various habitats including seagrass and macroalgae on rocks up to 35 meters depth [26], [28]. Specimens collected for this study were taken from two stations that represent different habitats of soritids in the Gulf (Figures 1 and 2): 1. Tur Yam characterized by *Halophila stipulacea* seagrass meadow, south of the port of Elat (N29 31.042 E034 55.590), at a water depth of 5 meters. 2. The Interuniversity Institute of Marine Sciences in Elat (IUI) characterized by pebbles at water depths of 5 meters and less than 2 meters (N29 30.653 E034 55.454).

## Specimen Collection

Living specimens of the soritids were collected from various depths along the Israeli Mediterranean coast and the Gulf of Elat, under the official approval of Israel Nature and Parks Authority (Figure 1, Table 1). The morphological distinction of the species followed the taxonomical conception of [29]. Specimens were collected alive with the substrate they lived on (seagrass or pebbles, Figure 2). In the laboratory, each specimen was examined under the binocular in order to see the algal symbionts and the pseudopods emerging from the thickened peristomal-rimmed apertures at the peripheral margin, confirming that the specimen is indeed alive. The living foraminifera were cleaned of food remains, sand and algae by a delicate brush and photographed with a digital microscope color camera (Leica, DFC290HD).

**Table 1:** Sampling locations, depths, habitats and abundances of live and dead Soritids that were found in the Mediterranean and Gulf of Elat.

		Station/Sampling year	Coordinates	WaterDepth (m)	Habitat
Live specimens	Gulf of Elat	Tur Yam (2011–2012)^1^	N29 31.04 E34 55.59	5	*Halophila stipulacea* sea grass
		IUI (2011–2012)^1^	N29 30.65 E34 55.45	2	Pebbles and rocks
		IUI (2011–2012)^1^	N29 30.65 E34 55.45	5	Pebbles and rocks
	Mediterranean	Shikmona (2011–2012) 1 Soritids hotspot	N32 49.45 E34 57.27	0.2	Pebbles and rocks, abrasion platform
Recently dead specimens	Mediterranean	Akhziv (1999)^2, 3^	N33 04.15 E35 06.49	3–30	Carbonate rich-substrate (rocky or sandy) with macroalgae
		Akhziv (1998)^2^	N33 03.30 E35 04.23	41.5	Carbonate rich-substrate with macroalgae
		Dado, Haifa (1996)^2^	N32 47.03 E34 55.52	25	Sandy
		Dor (1998)^2^	N32 36 30 E34 54 17	15	Sandy
		Herzeliyya (1998)^2^	N32 08 09 E34 46 26	9	Sandy
		Shikmona (2003–2004) 4	N32 49.34 E34 57.15	1–1.5	Macroalgae: *Cystoseira* sp.
Fossil record	Mediterranean	Ashqelon (2000)^3, 5^, Borehole K20	N31 63.10 E34 50.77	69.5–84.5 mbsl	Carbonate rich-substrate (rocky or sandy) with macroalgae
		Ashqelon (2000)^3, 5^, Borehole K38	N31 62.53 E34 49.36	83.4–90.4 mbsl	Carbonate rich-substrate (rocky or sandy) with macroalgae
		Caesarea (1994)^6^, Core C	N32 30.06 E34 53.28	+0.86 to −2.29 mbsl	Sandy substrate probably with vegetation

m bsl = meters below sea level.

1 This study;

2 Hyams et al., 2002;

3 Lazar, 2007;

4 Gruber et al., 2007;

5 Avital, 2002;

6 Toueg, 1996.

We also reexamined the morphology of dead soritids specimens that were collected from recent sediments along the Israeli Mediterranean shelf in previous studies [6], [22], [30] in order to estimate whether a morphological variation can be detected between recent and fossil soritids (Table 1).

### DNA Extraction, Amplification and Sequencing

The DNA was extracted from single specimens using guanidinium buffer as described in [31]. Part of the test was kept for the morphological documentation using a Scanning Electron Microscope (SEM). For each genetic type, multiple specimens were sequenced.

A fragment of the SSU rDNA (∼800 bp) was amplified by semi nested PCR in two overlapping fragments. For the first PCR 1 µl of the purified DNA was used in a total volume of 50 µl, with primers s14F3-NewB (Table 2). Thermal cycles used were: 30 seconds at 98°C, 5 seconds at 98°C, 20 seconds at 60°C and 30 seconds at 72°C. This cycle was repeated 35 times. The last cycle was followed by 5 minutes at 72°C for final elongation. For the second PCR, 1 µl of the first PCR product was used in a volume of 50 µl with primers s14F1-NewB (Table 2) and the same thermal cycles as the first PCR.

**Table 2:** PCR primers used for amplification and reamplification of soritid SSU genes and Symbiodinium ITS and LSU genes.

Name	Sequence	Forward	Reverse	Amplification	Reamplification
14F3	5′acgca(ac)gtgtgaaacttg	X		X	
14F1	5′aagggcaccacaagaacgc	X			X
New B	5′tgccttgttcgacttctc		X	X	X
S-Dino	5′cgctcctaccgattgagtga	X		X	
L0	5′gctatcctgag(ag)gaaacttcg		X	X	
ITS-Dino	5′gtgaattgcagaactcc	X			X

The amplified products were purified from gel and ligated into a pJET1.2 Plasmid using clone JET PCR cloning kit. Ligation mix was transformated into DH5α competent bacteria. Plasmids were extracted by Qiagen MiniPrep kit. For each specimen at least two clones were sequenced in order to look for variability within the specimen.

DNA amplification and reamplification of symbionts followed the same protocol as used for the PCR of soritid rRNA genes, primers are given in Table 2. A fragment of about 1100 bp comprising 5.8S rDNA, ITS2 and partial LSU rDNA was obtained for dinoflagellate symbionts of different host extractions (Table 3). The amplified PCR products were purified using High Pure PCR Purification Kit (Roche Diagnostics), cloned with the TOPO TA Cloning Kit (Invitrogen) following the manufacturer’s instructions and transformed into competent *Escherichia coli*. Up to three *Symbiodinium* clones were sequenced for each host specimen. Sequencing reactions were performed using the BigDye Terminator v3.1 Cycle Sequencing Kit (Applied Biosystems) and analyzed on a 3130XL Genetic Analyzer (Applied Biosystems). The new sequences reported in this paper were deposited in the EMBL/GenBank database and their accession numbers are listed in Tables 3 and 4.

**Table 3:** Details on symbiont sequences obtained in this study.

Host species	Host isolate	Sampling locality	Nr. of sequenced clones	Symbiont type	Symbiont accession nr.
*A. hemprichii*	1363	Elat	2	F5	HF582847, HF582848
*A. hemprichii*	12985	IUI, Elat	3	F5	HF582823, HF582832, HF582833
*A. hemprichii*	12986	IUI, Elat	2	F2, F5	HF582801, HF582821
*S. orbiculus*	29	Shikmona	3	F5	HF582834, HF582835, HF582836
*S. orbiculus*	55	Shikmona	3	F5	HF582837, HF582838, HF582839
*S. orbiculus*	65	Shikmona	2	F5	HF582840, HF582841
*S. orbiculus*	70	Shikmona	3	F5	HF582822, HF582830, HF582831
*S. orbiculus*	71	Shikmona	1	F5	HF582842
*S. orbiculus*	72	Shikmona	1	F5	HF582843
*S. orbiculus*	73	Shikmona,	3	F5	HF582844, HF582845, HF582846
*S. orbiculus*	206	Safaga, Red Sea	3	F2	HF582798, HF582799, HF582800
*S. orbiculus*	1283	Elat	2	F5	HF582849, HF582850
*S. orbiculus*	1284	Elat	3	F5	HF582851, HF582852, HF582853
*S. orbiculus*	13461	Tur Yam, Elat	2	F5	HF582820, HF582824
*S. orbiculus*	13464	Tur Yam, Elat	3	F5	HF582825, HF582826, HF582827
*S. orbiculus*	13467	Tur Yam, Elat	2	F5	HF582828, HF582829
*S. variabilis*	1275	Elat	2	F2	HF582805, HF582808
*S. variabilis*	12979	IUI, Elat	3	F2	HF582809, HF582810, HF582811
*S. variabilis*	12981	IUI, Elat	1	F2	HF582812
*S. variabilis*	12982	IUI, Elat	1	F2	HF582813
*S. variabilis*	13473	IUI, Elat	2	F2	HF582814, HF582815
*S. variabilis*	13478	IUI, Elat	3	F2	HF582816, HF582817, HF582818
*S. variabilis*	13479	IUI, Elat	3	F2	HF582802, HF582803, HF582804
*S. variabilis*	13480	IUI, Elat	2	F2	HF582806, HF582807,
*S. variabilis*	13481	IUI, Elat	1	F2	HF582819

**Table 4:** Details on soritid sequences obtained in this study and published data.

Species	Isolate	Sampling locality	Samplingdepth (m)	Habitat	Nr. of sequenced clones	Accession nr.	Haplotype
*Amphisorus hemprichii*	12987	IUI, Elat	5	Pebbles	2	HF582900, HF582903	
*Amphisorus hemprichii*	12985	IUI, Elat	5	Pebbles	1	HF582901	
*Amphisorus hemprichii*	12986	IUI, Elat	5	Pebbles	1	HF582902	
*Amphisorus hemprichii*	26	Tur Yam, Elat	5	Seagrass	1	HF582904	
*Amphisorus hemprichii*	92	Tur Yam, Elat	5	Seagrass	1	HF582905	
*Amphisorus hemprichii*	87	Tur Yam, Elat	5	Seagrass	3	HF582906, HF582907, HF582908	
*Amphisorus hemprichii*	1305	Taba	not identified	not identified	1	AJ278052	
*Amphisorus hemprichii*	1366	Elat	not identified	not identified	1	AJ278048	
*Amphisorus hemprichii*	1361	Elat	not identified	not identified	1	AJ404315	
*Amphisorus hemprichii*	1363	Taba	not identified	not identified	1	AJ278049	
*Amphisorus kudakajimaensis*	1635	Guam	not identified	not identified	1	AJ404316	
*Amphisorus kudakajimaensis*	1636	Guam	not identified	not identified	1	AJ278050	
*Amphisorus kudakajimaensis*	337	Japan: Sesoko	not identified	not identified	1	AJ278051	
*Marginopora vertebralis*	500	Australia: Lizard Island	not identified	not identified	1	AJ278058	
*Marginopora vertebralis*	478	Australia: Lizard Island	not identified	not identified	1	AJ278060	
*Marginopora vertebralis*	499	Australia: Lizard Island	not identified	not identified	1	AJ404312	
*Marginopora vertebralis*	1610	Guam	not identified	not identified	1	AJ278059	
*Marginopora vertebralis*	1686	Guam	not identified	not identified	1	AJ278062	
*Marginopora vertebralis*	1645	Guam	not identified	not identified	1	AJ278061	
*Parasorites* sp.	1634	Guam	not identified	not identified	1	AJ404305	
*Sorites orbiculus*	13464	Tur Yam, Elat	5	Seagrass	2	HF582855, HF582856	H2
*Sorites orbiculus*	13467	Tur Yam, Elat	5	Seagrass	1	HF582857	H2
*Sorites orbiculus*	13461	Tur Yam, Elat	5	Seagrass	1	HF582858	H2
*Sorites orbiculus*	29	Shikmona	0.2	Pebbles	3	HF582859, HF582864, HF582865	H1
*Sorites orbiculus*	55	Shikmona	0.2	Pebbles	3	HF582860, HF582862 HF582866	H1
*Sorites orbiculus*	85	IUI, Elat	2	Pebbles	2	HF582861 HF582879	H1
*Sorites orbiculus*	65	Shikmona	0.2	Pebbles	3	HF582863 HF582867, HF582868	H1
*Sorites orbiculus*	70	Shikmona	0.2	Pebbles	1	HF582869	H1
*Sorites orbiculus*	71	Shikmona	0.2	Pebbles	3	HF582870 HF582871 HF582880	H1
*Sorites orbiculus*	72	Shikmona	0.2	Pebbles	1	HF582872	H1
*Sorites orbiculus*	73	Shikmona	0.2	Pebbles	1	HF582873	H1
*Sorites orbiculus*	76	Shikmona	0.2	Pebbles	2	HF582874 HF582875	H1
*Sorites orbiculus*	80	IUI, Elat	2	Pebbles	2	HF582876 HF582881	H1
*Sorites orbiculus*	81	IUI, Elat	2	Pebbles	2	HF582877 HF582882	H1
*Sorites orbiculus*	84	IUI, Elat	2	Pebbles	2	HF582878 HF582885	H1
*Sorites orbiculus*	82	IUI, Elat	2	Pebbles	1	HF582883	H1
*Sorites orbiculus*	83	IUI, Elat	2	Pebbles	1	HF582884	H1
*Sorites orbiculus*	1678	Guam	not identified	not identified	1	AJ278055	H4
*Sorites orbiculus*	1629	Guam	not identified	not identified	1	AJ278053	H3
*Sorites orbiculus*	751a	USA: Florida Keys	not identified	not identified	1	AJ278056	H3
*Sorites orbiculus*	1282	Elat	not identified	not identified	1	AJ278057	H1
*Sorites orbiculus*	1305	Taba	not identified	not identified	1	AJ404310	H2
*Sorites* sp.	1591a	Guam	not identified	not identified	1	AJ278054	
*Sorites* sp.	489	Australia:Lizard Island	not identified	not identified	1	AJ404309	
*Sorites* sp.	1368	Elat	not identified	not identified	1	AJ404311	
*Sorites* sp.	836	Florida Keys	not identified	not identified	1	AJ404308	
*Sorites variabilis*	12982	IUI, Elat	5	Pebbles	2	HF582887 HF582888	
*Sorites variabilis*	13472	IUI, Elat	5	Pebbles	1	HF582889	
*Sorites variabilis*	13480	IUI, Elat	5	Pebbles	2	HF582890 HF582896	
*Sorites variabilis*	13478	IUI, Elat	5	Pebbles	1	HF582891	
*Sorites variabilis*	13479	IUI, Elat	5	Pebbles	1	HF582892	
*Sorites variabilis*	13481	IUI, Elat	5	Pebbles	1	HF582893	
*Sorites variabilis*	13477	IUI, Elat	5	Pebbles	1	HF582894	
*Sorites variabilis*	13473	IUI, Elat	5	Pebbles	1	HF582895	
*Sorites variabilis*	12979	IUI, Elat	5	Pebbles	3	HF582897, HF582886 HF582898	
*Sorites variabilis*	12981	IUI, Elat	5	Pebbles	1	HF582899	
*Sorites variabilis*	1333	Elat	not identified	not identified	1	AJ404313	

### Sequence Analyses

**Figure 3:**
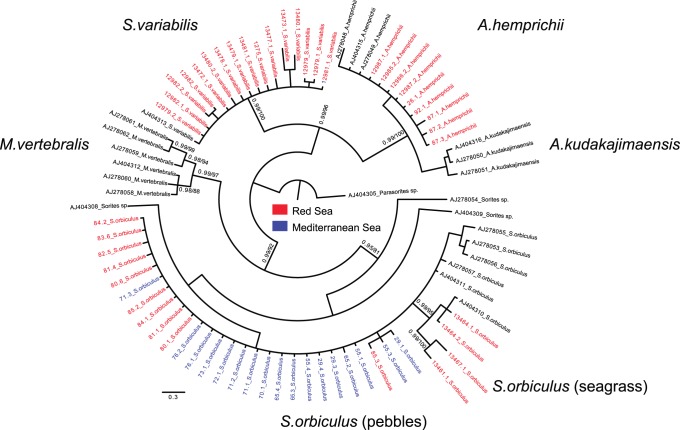
Bayesian phylogenetic tree (GTR+I+Γ) showing the phylogenetic position of 72 partial SSU 18S rDNA of soritids. The 52 sequences obtained for this study are marked by their DNA isolate numbers. Numbers at nodes indicate (from left to right) posterior probabilities (BI) and bootstrap values (ML). The tree is rooted on Parasorites sp.

The soritids sequences were automatically aligned with soritids sequences from NCBI GenBank (published by [27]) using Seaview 4.3.3. [32]. Phylogenetic analysis was based on the maximum likelihood (ML) method using PhyML3.0 program [33] under the GTR+G model based on MEGA5 [34]. The Bayesian analysis was performed with MrBayes 3.1.2 [35]. The tree was rooted on *Parasorites* Douville the closest sister group to Soritinae [27] (Figure 3). The analysis consisted of four simultaneous chains that were run for 1,100,000 generations, and 4400 trees were sampled, 1100 of which were discarded as burn-in. Posterior probabilities at all nodes were estimated for the remaining trees.

**Figure 4:**
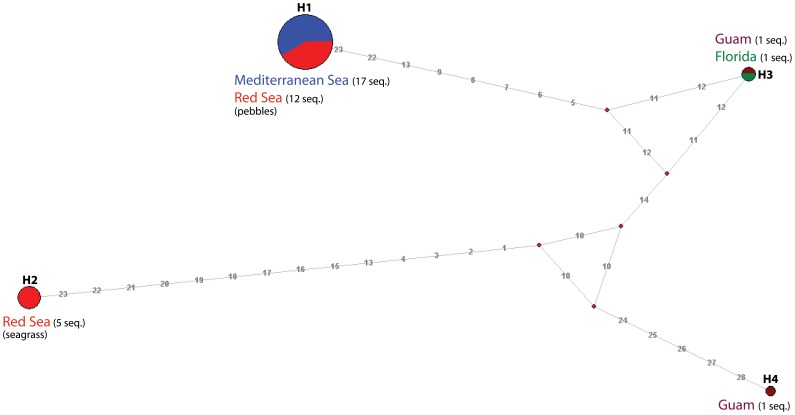
Median joining network for four haplotypes of Sorites orbiculus based on 37 sequences. Numbers in light grey correspond to mutated positions. Circle sizes of haplotypes H1 to H4 are proportional to the number of identical sequences they contain. Each color corresponds to a different locality. Red dots indicate missing haplotypes (extinct or not sampled).

Median joining network was drawn using Network software (www.fluxus-engineering.com) according to [36]. The analysis was based on all mutations (indels and substitutions) of *S. orbiculus* (Figure 4). In the first run all mutations were equally weighted. In the second run sites were weighted inversely proportional to their frequency in order to take account of recurrent mutations.

**Figure 5:**
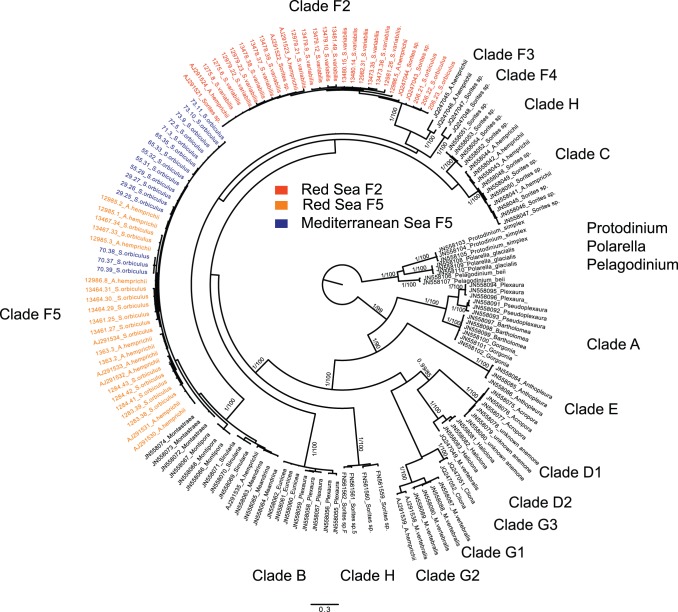
Bayesian phylogenetic tree (GTR+G model) showing the phylogenetic position of 140 partial 5.8S rDNA, ITS2 and LSU rDNA sequences of *Symbiodinium*. The 56 sequences obtained for this study are marked by their DNA isolate numbers. Numbers at nodes indicate (from left to right) posterior probabilities (BI) and bootstrap values (ML). The tree was rooted with *Protodinium*, *Polarella* and *Pelagodinium*.

The *Symbiodinium* sequences were aligned to an existing database using Seaview 4.3.3. [32]. Based on MEGA5 [34], a GTR+G model of evolutionary changes was selected. A phylogenetic tree was constructed using RAxML as implemented in BlackBox [37] (Figure 5). Bayesian analysis was performed with MrBayes 3.2.1 [35]. The analysis consisted of four simultaneous chains that were run for 10,000,000 generations, and 10,000 trees were sampled, 2000 of which were discarded as burn-in. Posterior probabilities at all nodes were estimated for the remaining trees.

## Results

### Habitat Characterization and Morphological Distinctions

**Figure 6:**
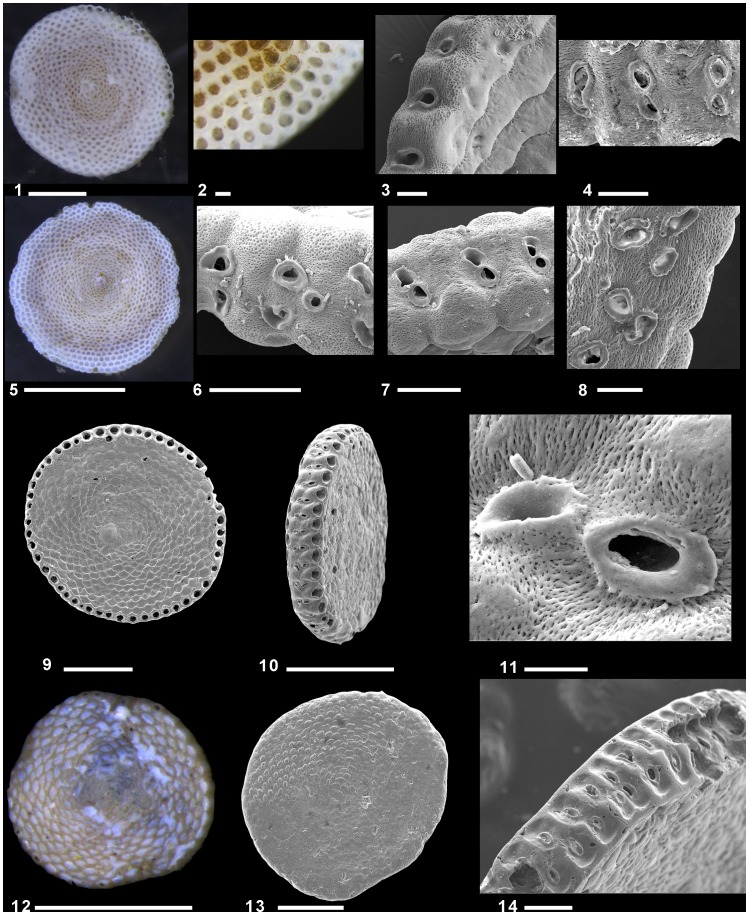
Specimens of Sorites orbiculus Forskål from the Gulf of Elat and the Eastern Mediterranean Israeli coast, showing the large variability of apertural shapes and arrangement among specimens and also within single specimens. 1–4. Gulf of Elat: 1. Lateral view of live individual (IUI, 2 m, rocks/pebbles), digital camera, scale 500 µm; 2. Details of chambers with symbionts, digital camera, scale 100 µm; 3. Oblique apertural view (Tur Yam, 5 m, seagrass), SEM, scale 20 µm; 4. Oblique apertural face showing variation in apertural shapes from a single row of 8-shaphed aperture to two separate aperture rows, in early growth stage bordered with a thickened peristomal rim, SEM, scale 100 µm; 5–8. Shikmona (0.5–1.5 m). 5. Lateral view of live individual 0.2 m, rocks/pebbles, digital camera, scale 500 µm; 6. Detailed apertural face with pitting and two separate rows of apertures in alternating radial position and auxiliary aperture, SEM, scale 100 µm; 7. Apertural face in early growth stage with 8-shaped apertures, SEM, scale 100 µm; 8. Apertural face showing a late growth stage with separated apertures, each with complete, circular or irregularly folded peristome, Gruber et al., 2007, SEM, scale 50 µm; 9–11. Transect C-6, Caesarea (1.29 m below surface) fossil material (Toueg, 1996), SEM. 9. Lateral view, scale 500 µm; 10. Oblique profile view, scale 500 µm; 11. Detail of Fig. 10, scale 20 µm; 12–14. Borehole K-20 Ashqelon, fossil material (Avital, 2002, Lazar, 2007): 12–13. 63.95 m below top core; 12. Lateral view, digital camera, scale 1 mm; 13. Lateral view, SEM, scale 500 µm; 14. Oblique profile view showing two rows of oval apertures with peristomal rims (65.5 m below surface), SEM scale 100 µm.

**Figure 7:**
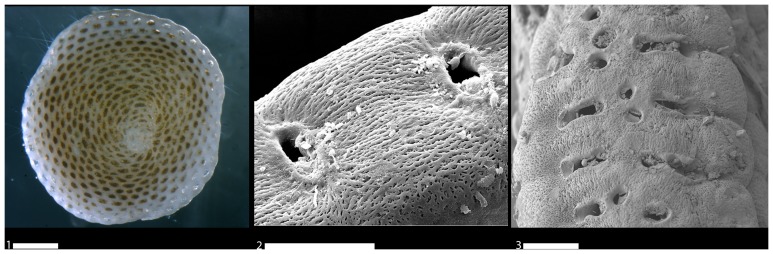
Specimens of *Sorites variabilis* and *Amphisorus hemprichii* showing a faint rim around the apertures. 1–2. *S. variabilis.* 1. Lateral view of live specimen, digital camera, scale 100 µm; 2. Apertural face with faint rim around the apertures, SEM scale 50 µm; 3. Apertural face of *A. hemprichii* with faint rim around the apertures, SEM scale 50 µm.

Living specimens of *A. hemprichii* and *S. variabilis* were found only in the Gulf of Elat attached to rock pebbles at variable water depths (typically >1 m) or to *Halophila* leaves or stalks. *Sorites variabilis* mainly differs from *A. hemprichii* by having a much thinner test (±120 µm) and a single row of apertures. Both *S. variabilis* and *A. hemprichii* are morphologically distinguishable from *S. orbiculus* by the appearance of faint or no rim surrounding their apertures (Figures 6–7).

*Sorites orbiculus* was found alive in the Gulf of Elat as well as in high numbers in Shikmona. In Elat, it was found attached to rock pebbles at water depths of about 1 m or attached to *H. stipulacea* leaves. It is slightly thicker than *S. variabilis* (±160 µm) and has apertures surrounded by a distinct rim (Figure 6). Specimens of this species show a great variability in the shape and number of rows of apertures. They appear either in one or two rows, occasionally two are fused into one large, 8 shaped aperture. In some specimens auxiliary apertures are seen (Figure 6).

Morphological identification of the specimens collected in Shikmona, indicated that they all belong to the species *S. orbiculus* (Figure 6). No morphological difference was found between specimens of *S. orbiculus* from different habitats (seagrass and pebbles) from the Gulf of Elat, the living specimens from Shikmona and the fossils specimens from the Mediterranean.

### Genetic Analysis of the Foraminifera

A fragment of the SSU rDNA, (800 basepairs) was obtained from 32 specimens of the morpho species *S. orbiculus*, *S. variabilis* and *A. hemprichii* collected from the different stations in the Gulf of Elat and Shikmona (Figure 3, Table 4). The obtained sequences were compared to sequences from the NCBI GenBank of 27 specimens of the three species plus *Marginopora vertebralis* and *Parasorites* sp., used as outgroup (Table 4). The phylogenetic tree constructed from these sequences shows that the soritids cluster into two main clades: *S. variabilis/A. hemprichii* and *M. vertebralis/Sorites* spp. (92% BV and 96% BV, respectively). The *S. variabilis/A. hemprichii* clade is divided into two distinct subclades corresponding to *A. hemprichii* and *S. variabilis* (each supported by 100% BV), that were found only in Elat. The *M. vertebralis/Sorites* spp. clade comprises two distinct clades *M. vertebralis* and *S. orbiculus* (97% BV and 81% BV, respectively).

As the phylogenetic analyses of soritids do not show a significant support of the *S. orbiculus* clade we added a population genetic analysis to investigate intraspecific relationships in this species (Figure 4) as suggested for such cases by [38]. The network was drawn using all sequences (37) of *S. orbiculus* with a total of 28 mutated sites. Sequences present in each haplotype are reported in Table 4. Our results indicate that *S. orbiculus* splits into four different haplotypes. Haplotypes H1 and H3 each occur in two different localities. The haplotypes H2 and H4 are present in one locality only.

Specimens of *S. orbiculus* from the Gulf of Elat and Shikmona split into two distinct haplotypes, each one present in a different type of habitat: shallow water pebbles (H1) and the seaweed *H. stipulacea* (H2). All specimens of *S. orbiculus* from Shikmona were found to be genetically identical to the specimens from the shallow water pebbles at Elat (H1).

### Genetic Analysis of the Algal Symbionts

Congruent results are obtained by RAxML and Bayesian analyses of combined partial ITS and LSU rDNA sequences of *Symbiodinium*. The tree comprises 14 symbiont clades and is rooted in the free-living dinoflagellate genera *Protodinium*, *Polarella* and *Pelagodinium* (Figure 5). The 56 symbiont sequences of soritids from the Red and Mediterranean Seas obtained for this study are belonging either to clade F2 or F5, which are both well supported (100% BV, 1PP). All symbionts of *S. variabilis* belong to the F2 clade while symbionts from *S. orbiculus* and *A. hemprichii* are assigned either to the clade F2 or the clade F5. One specimen of *A. hemprichii* (isolate nr. 12986) hosts both types of symbionts. All symbionts from *S. orbiculus* collected in the Mediterranean Sea branch within the F5 clade.

Clade A branches at the base of all other symbiont clades, followed by clades E, D1, D2, G3, G1, G2, H, B and C. Clade C is branching off a large group containing F2, F3 and F4 that are all sisters to F5. Most of the symbiont clades are specific to foraminifera (G1, G2, H, F2, F3, F4, C). Only two clades are present in foraminifera as well as cnidarians (D2, F5) and four symbiont types are characteristic for cnidarians (A, E, D1, G3).

## Discussion: Evidence for Lessepsian Invasion of Soritids

Recent studies by Langer and colleagues [8], [13], [15] provided the most comprehensive biogeographical documentation of the expansion of larger benthic foraminifera (LBF) in the Mediterranean. In general, the abundance and species richness of LBF are increasing steadily in the Eastern Mediterranean Sea. This increase has been attributed to the hyper-oligotrophy together with the increasing temperatures that allow the LBF to settle and thrive and often take over the native fauna in many ecological niches, as documented in the shallow rocky environments of the Israeli Mediterranean inner shelf [6], [22], [30]. Some of these species have now become extremely abundant in these environments. It is assumed that the expansion of LBF in the Mediterranean is a consequence of the opening of the Suez Canal, which promoted a unidirectional invasion either by natural dispersal or by anthropogenic factors [8]. Yet, some fossil LBF species are known from subsurface Pleistocene marine sediments that were recovered from the Israeli coastal plain and inner shelf [22], [39] (Figure 6). Their occurrences imply that these species originated from a tropical Atlantic source and colonized the Eastern Mediterranean mainly during warmer glacial and warm interglacial periods [22]. This raises the question whether the recent populations of LBF observed in the studied hotspot represents reviving of native Mediterranean fauna, recent invasion from the Atlantic or a pure introduction of new Lessepsian invaders.

Because the morphological variability observed within the specimens of soritids investigated in this study was found to be greatly inconsistent, making it impossible to distinguish living, dead and fossil specimens based on traditional taxonomic features (Figure 6), we tackled this question by comparing the genotypes of soritid foraminifers found in the Eastern Mediterranean and the Red Sea. Our results illustrated in the phylogenetic tree of the soritids (Figure 3) and in the network analysis (Figure 4) clearly show that the population of *S. orbiculus* found in Shikmona is genetically identical to one of the soritid haplotypes identified in the Gulf of Elat and therefore seems to be Lessepsian. Specifically, the Shikmona specimens are genetically identical to those of *S. orbiculus* collected from pebbles at very shallow water depths in the Gulf of Elat (H1, Figure 4). Our results also demonstrate that the population in Shikmona retained the same ecological/habitat preferences as in the Red Sea (Figure 2, Figure 4, Table 4).

The *S. orbiculus* clade consists of great number of highly divergent haplotypes [27], [40], which demonstrates that the seemingly cosmopolitan distribution of this species is an artifact of morphological conservation. The observed branching between *S. orbiculus* from the *Halophila* leaves and the pebbles in IUI and Shikmona (Figure 4) indicates that genetic divergence also exists between habitats and not only between different geographic regions. The divergence between the shallow water pebbles population of IUI and Shikmona from those of Florida and Guam indicates that they are genetically separated and therefore are not directly related (Figure 4). It is further reasonable to postulate that if the population in Shikmona is a relict of a native Mediterranean source (i.e. post Messinian Atlantic origin) it should have been branched separately from the modern Red Sea population.

It is important to note that not all Red Sea soritids successfully invaded the Mediterranean ([8] and compare with [29]). A distinct selectivity in the invasion process suggests that the environmental differences between the two seas act as a filter allowing only certain haplotypes to settle in the Eastern Mediterranean. This is supported by the fact that no specimens of the two morphologically distinctive species *S. variabilis* and *A. hemprichii* were found alive at the various sites along the coast of Israel or elsewhere in the Mediterranean [3]. Our combined genetic and morphological analyses revealed that these species share the morphological feature of apertures with faint rim that signifies their evolutionary branching from the lineage of *S. orbiculus* (Figures 3, 7). This finding indicates that the genus *Sorites* is polyphyletic and consequently *S. variabilis* should be amended as species of the genus *Amphisorus*. The common evolutionary history of *S. variabilis* and *A. hemprichii* implies that they may share some physiological constrains that presently limit their ability to successfully invade the Mediterranean.

Previous reports show that *S. orbiculus* occupies the entire eastern Levantine Basin, as well as the Adriatic and Tyrrhenian seas [8], [13], [41]. Its distribution was attributed to the ability to tolerate temperatures below 14°C [13]. The very shallow depth habitats of *S. orbiculus* recognized in this study imply that it may also tolerate temperatures beyond the typical range of this group. Our results do not rule out the possible existence of other *S. orbiculus* haplotypes somewhere else in the Mediterranean, but it does prove dominance of a single invasive haplotype in the Israeli coast.

The selectivity of invasive species also concerns their symbionts. All *Symbiodinium* from *S. orbiculus* collected in Shikmona belong to the F5 clade. Interestingly, the *Symbiodinium* phylotype F2, endemic to the Red Sea has not been found in the specimens from Shikmona. Clade F5 was also recognized from the *S. orbiculus* haplotype collected from the shallow water pebbles in the Gulf of Elat (Figure 5, Table 3). This clade is known to dominate in the Red Sea and also has been found in the Indian Ocean and the Caribbean Sea [40]. Its worldwide distribution may reflect easier adaptation to different environmental conditions, which certainly helps the migration to new areas.

## Conclusions

Our study shows the potential of using LBF in combined genetic and morphological studies to characterize a Lessepsian invasion. It provides the first genetic and ecological evidence that the modern Eastern Mediterranean population of *S. orbiculus* is likely Lessepsian; it occupies very similar habitats in both regions, and keeps their original symbionts. The mechanism responsible for the migration of the *S. orbiculus* population from the Red Sea and its establishment in Shikmona could be due to either natural dispersal or anthropogenic factors. A further study is needed to conclude if all the Mediterranean populations of *S. orbiculus* originated from the same source of genotype that was identified in the present study or whether there are some areas that still contain the native fauna or other Red Sea haplotypes.
